# Pregnancy in Inflammatory Bowel Disease: Data from a Real-World Cohort in Germany

**DOI:** 10.3390/jcm13247710

**Published:** 2024-12-17

**Authors:** Mousa Ayoub, Anna Muzalyova, Alanna Ebigbo, Sandra Nagl, Christoph Römmele, Johanna Classen, Julia Wanzl, Carola Fleischmann, Sami Ayoub, Vidan Tadic, Jakob Schlottmann, Elisabeth Schnoy

**Affiliations:** 1 Internal Medicine III, University Hospital Augsburg, 86156 Augsburg, Germany; alanna.ebigbo@uk-augsburg.de (A.E.); sandra.nagl@uk-augsburg.de (S.N.); christoph.roemmele@uk-augsburg.de (C.R.); johanna-maria.classen@uk-augsburg.de (J.C.); julia.wanzl@uk-augsburg.de (J.W.); vidan.tadic@uk-augsburg.de (V.T.); jakob.schlottmann@uk-augsburg.de (J.S.); 2 Digital Medicine, University Hospital Augsburg, 86156 Augsburg, Germany; anna.muzalyova@uk-augsburg.de; 3 Department of Gastroenterology, Hepatology and Endocrinology, Klinikum Nürnberg, 90419 Nuremberg, Germany; carola.fleischmann@klinikum-nuernberg.de; 4 Internal Medicine I, University Hospital Augsburg, 86156 Augsburg, Germany; sami.ayoub@uk-augsburg.de

**Keywords:** inflammatory bowel disease, pregnancy, ulcerative colitis, Crohn’s disease

## Abstract

**Background**: Inflammatory bowel disease (IBD) frequently manifests at a young age, during the peak fertility years. Understanding the risks of negative pregnancy outcomes associated with IBD is crucial for effective pregnancy management and support. Additionally, it is essential to provide patients with the necessary knowledge to make informed choices and foster their confidence in navigating pregnancy while maintaining effective disease management. Although IBD frequently appears during the peak fertility years, knowledge about managing pregnancy in the context of IBD remains limited and often inaccurate among both physicians and patients. Our study aims to assess the complications occurring during pregnancy in patients with IBD, considering the level of disease activity, and to evaluate the standard of care provided to patients with chronic inflammatory conditions through a cohort analysis. **Methods**: Patients with IBD who had children were included in this single-center mixed-method (retrospective and prospective) study. Clinical data, disease progression, course of pregnancy, and complications were examined in women. Outcomes for children of men with IBD were also analyzed. To supplement the data, a survey addressing various pregnancy-related topics, including all patients from the university outpatient clinic for IBD, was conducted over a period of six months. **Results**: A total of 410 patients were screened retrospectively between 2010 and 2021. In total, 134 patients who had children were included in the study: 51.4% (*n* = 69) had Crohn’s disease, 44% (*n* = 59) had ulcerative colitis, and 4.6% (*n* = 6) had unclassified inflammatory bowel disease. Of the women, 85% (*n* = 34) were in remission for at least three months before pregnancy, 14.6% (*n* = 6) experienced an acute flare-up during pregnancy, and 10.3% (*n* = 4) and 7.7% (*n* = 3) had active disease at the time of delivery and during breastfeeding, respectively. Patients with IBD who were in remission before pregnancy did not experience a higher risk of pregnancy complications (no cases of pre-eclampsia or placental abruption were reported in this group). However, the rates of gestational diabetes and fever during pregnancy were 10% for those in remission, compared to 25% for those with active disease. **Conclusions**: Patients with IBD in remission did not present an increased risk of pregnancy complications. However, our survey indicates that those with active disease at conception were more likely to experience complications such as gestational diabetes and fever. These findings underscore the importance of adequate patient education regarding the safety of various IBD medications during pregnancy in order to avoid pregnancy-related complications.

## 1. Introduction

Inflammatory bowel diseases (IBDs)—including Crohn’s disease (CD) and ulcerative colitis (UC)—are complex disorders of the digestive tract characterized by chronic inflammation. These diseases are a growing global health concern, with increasing incidence worldwide [1]. They significantly affect the quality of life of patients and place a considerable burden on healthcare systems. Despite extensive research efforts, the precise causes of IBDs remain only partially understood. It is believed that a combination of genetic, immunological, and environmental factors contributes to the development of these diseases [2].

A total of 50% of IBD patients are diagnosed before the age of 35 [3]. Women in this age group are often in their peak fertility years and may be planning pregnancies. For this patient group, the potential impact of pregnancy on IBD activity is of particular interest, especially in the context of family planning [4]. Patients with IBD worry about the activity of their disease during pregnancy and the potential complications for their children. Additionally, female patients have concerns about the use of IBD medications during pregnancy, fearing that these drugs might adversely affect their children. These issues have significantly influenced the family planning decisions of numerous women diagnosed with IBD over the past five decades [5].

A survey focusing on women diagnosed with IBD indicated that 46% of the respondents experienced a shift in their perspective on childbearing due to IBD. Moreover, 16% of these women opted not to have children—a number significantly higher than the 6.2% observed in the general population [3,4,6]. Disease activity at the time of conception is an important predictor of the disease course during pregnancy and pregnancy outcomes. If the IBD is inactive at the time of conception, the likelihood of a disease flare is comparable to that in non-pregnant patients [7,8]. Conversely, if the disease is active at conception, there is a 60% chance that the disease will remain active or worsen during pregnancy [9,10].

Several IBD medications show no discernible negative effects on pregnancy or fertility. Moskovitz et al. studied 207 medications in 113 pregnant IBD patients, and they found that many IBD medications had no significant side effects on pregnancy outcomes [11]. Nevertheless, most patients—mostly due to a lack of effective medical advice before and during pregnancy—fear the side effects of IBD medications and their impacts on the child.

Several studies have reported that mothers with IBD have a higher risk of preterm birth or stillbirth; however, it has not been conclusively proven that IBD with controlled disease activity can increase the risk of preterm birth or stillbirth [12,13,14]. Men with IBD also worry that their disease could negatively affect their children, as well as their fertility.

Given the advancements in the management of IBD in recent decades, concerns regarding the impacts of pregnancy on disease activity may be overstated [15]. To provide further insight into the correlation between IBD activity and adverse events during pregnancy, we assessed the pregnancy outcomes in IBD patients in relation to disease activity in a cohort analysis for this single-center study. In addition, we assessed patient perspectives using a structured questionnaire focused on pregnancy-related issues in IBD patients. This tool included demographic details, disease and treatment history, pregnancy outcomes, and patient experiences with pre-pregnancy counseling, allowing for systematic data collection to assess the impacts of IBD on pregnancy, allowing us to gain more comprehensive insights into their experiences and concerns (in Supplementary Materials).

## 2. Materials and Methods

### 2.1. Study Design

Our study involves both a retrospective data analysis and a prospective cohort questionnaire analysis, conducted at the Department of Gastroenterology, University Hospital of Augsburg, in southern Germany.

### 2.2. Patient Selection

All IBD patients in our department between 2010 and 2021 were screened. We included female and male patients who had become pregnant or fathered a child. The IBD patients in our clinic were exclusively cared for by physicians with advanced expertise in IBD. The inclusion criteria for participants required them to be over 18 years of age, have a confirmed diagnosis of IBD (CU, UC, or IBD-U), and provide written informed consent for participation in the study. Patients were excluded if they demonstrated a limited capacity to consent.

### 2.3. Data Collection

#### 2.3.1. Retrospective Analysis

We obtained data from available electronic medical records. The following parameters were recorded: demographic data (age, gender), comorbidities, age of diagnosis, disease duration, and pattern of involvement—for UC, involvement was categorized as pancolitis, left-sided colitis, or proctitis; for CD, involvement included the small bowel, colon (or both), or upper gastrointestinal tract. Additional data included hospital admissions, prior medical treatments (with details on duration, response, side effects, and discontinuation), steroid treatment (including duration), and any surgical treatments. Remission status, vaccination status during pregnancy, anemia, and biomarkers, including calprotectin and C-reactive protein (CRP), were also documented. We aimed to investigate whether pregnancy affects fecal calprotectin levels in order to test its reliability as a tool for assessing IBD activity during pregnancy [16], and to assess the impact of pregnancy on CRP levels in these patients [17]. We assessed disease activity and treatment during pregnancy, birth, and breastfeeding.

Other recorded factors included treatment modifications during pregnancy (and their reasons), pregnancy complications, flare-ups, duration of pregnancy, birth outcomes, and breastfeeding practices. For male participants, complications during their partner’s pregnancy and the health of their children were also considered.

#### 2.3.2. Prospective Survey

We also issued a prospective cohort survey addressing family planning choices among all patients in our outpatient IBD clinic. In addition to the previously listed parameters, participants provided information on their family planning choices, fertility treatments, family history, and additional maternal and newborn outcomes. These included ultrasound examination, routine 4-week pediatric examination, complications associated with breastfeeding, lifestyle changes during pregnancy, and advice received from physicians during pregnancy.

### 2.4. Disease Activity and Pregnancy Complications

Additionally, we investigated the correlations between disease activity and pregnancy complications. Pregnancy complications primarily included pre-eclampsia, gestational duration, gestational diabetes, fever during pregnancy, premature detachment of the placenta, mode of delivery, and birth weight. We selected these specific pregnancy complications based on their frequency in the general population, in order to assess whether they occurred more frequently in patients with IBD [18].

While all flares in each patient were considered as a single variable, we analyzed pregnancy complications individually to examine their individual impact on IBD patients. We performed a disease activity score analysis on IBD patients who completed the prospective questionnaires. Relevant data for calculating the disease activity scores were collected in 21 of 80 surveyed female patients who provided the necessary information.

We used the Crohn’s Disease Activity Index (CDAI) for patients with CD [19], where a score above 150 indicates active disease. For the CDAI, interpreting and quantifying symptoms such as “liquid stools” was challenging because they are difficult to define precisely. As a result, we estimated stool frequency using data from the questionnaires, recognizing the inherent difficulties in accurately capturing daily symptom variability. Moreover, extraintestinal manifestations were recorded, including arthritis/arthralgias, iritis/uveitis, and skin manifestations. This score was calculated for 12 patients with Crohn’s disease. These limitations are clearly stated to provide transparency and context to the findings presented (Table S1).

The Mayo score was intended for patients with UC. For the Mayo score, it was not possible to obtain endoscopic findings close to the time of conception in all patients. Therefore, we calculated the partial Mayo score, which excludes the endoscopic component, for 9 patients with UC. This approach allowed us to continue to evaluate disease activity for patients with UC, where scores of 0–1 indicate remission and scores of 2–9 represent varying degrees of mild to severe activity [20] (Table S2).

This study was performed in accordance with Good Clinical Practice and the Declaration of Helsinki. It was approved by the Ethics Committee at the University of Regensburg, Regensburg, Germany (#23-3208-101).

### 2.5. Statistics

Patient demographics and baseline characteristics were summarized using descriptive statistics. Continuous variables related to the study population, medication, newborn outcomes, breastfeeding, and pregnancy complications are presented as medians with ranges, indicating the minimum and maximum sample values. For comparisons of birth outcomes in relation to remission status before pregnancy, continuous variables such as birth weight and gestational duration are expressed as means with standard deviations. Categorical variables are reported as absolute frequencies and percentages.

To assess the associations between categorical variables, Fisher’s exact test was applied when sample sizes were small; specifically, when any cell in the contingency table contained fewer than five observations. In all other cases, the Chi-squared test was used.

Interval-scaled variables were compared using the Mann–Whitney U-test for independent samples, with a significance level set at 0.05. Data management, along with descriptive and inferential statistical analyses, was performed using IBM SPSS (version 27), while graphics were generated in Excel (version 2303).

## 3. Results

### 3.1. Patient Demographics

#### 3.1.1. Retrospective Data (2010–2021)

We retrospectively analyzed a total of 410 patients diagnosed with inflammatory bowel disease (IBD) in the outpatient clinic of the University Hospital Augsburg.

Among these patients, 134 had children and were thus included in the study. This group consisted of 80 females (59.7%) and 54 males (40.3%), with a median age of 46.5 years (range: 27–85 years) at the time of the study. The initial diagnosis was made at a median age of 32 years (range: 9–77 years). Breakdown by disease type revealed 69 patients (51.4%) with Crohn’s disease (CD), 59 (44.0%) with ulcerative colitis (UC), and 6 (4.6%) with unclassified IBD (IBD-U). Additional retrospective data on patient characteristics, including clinical remission rates and smoking habits, are detailed in Table 1.

At the time of data collection, 91.25% (*n* = 73) of the included female patients and 66.7% (*n* = 36) of the male patients were in remission. We also investigated additional factors that could negatively affect pregnancy outcomes, such as alcohol and smoking.

Only 2.5% (*n* = 2) of the female patients and 5.6% (*n* = 3) of the male patients regularly consumed alcohol. Furthermore, 5.0% (*n* = 4) of the women and 1.9% (*n* = 1) of the men were regular smokers.

#### 3.1.2. Prospective Data (From 2023)

For a subset of these patients, prospective data were collected from May 2023 onward. This follow-up allowed us to capture any recent changes or emerging trends within the demographic profile, especially as they relate to ongoing disease activity and lifestyle adjustments. The prospective data supplemented the retrospective findings by highlighting current remission rates and any updated demographic information.

All study population data are listed in Table 1.

### 3.2. Disease Characteristics

The median duration from disease onset to the first pregnancy was 6 years. At the time of data collection (cut-off 12/2023), 109 out of 134 (83.9%) patients were in remission under their current therapy. Furthermore, 24.5% had a positive family history of IBD, and 45 out of 134 (33.5%) patients underwent surgery due to their inflammatory bowel disease. The most common surgery performed was ileocecal resection, followed by surgical incision of anal abscesses. Hemicolectomy and partial small intestine resection were also reported (see Table 2).

**Figure 1:**
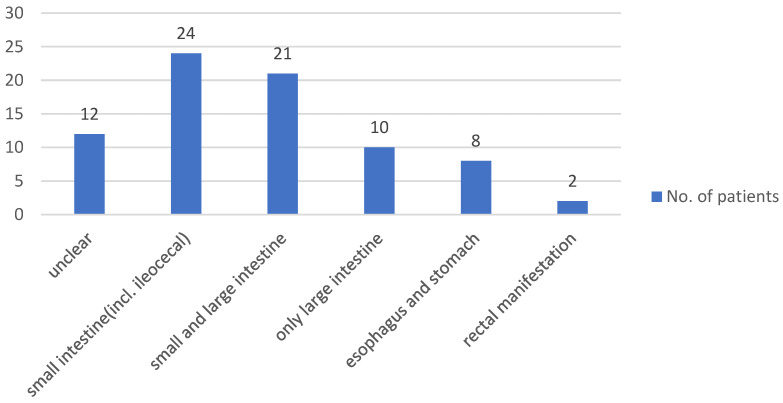
Distribution of disease patterns in CD patients in the study cohort.

**Figure 2:**
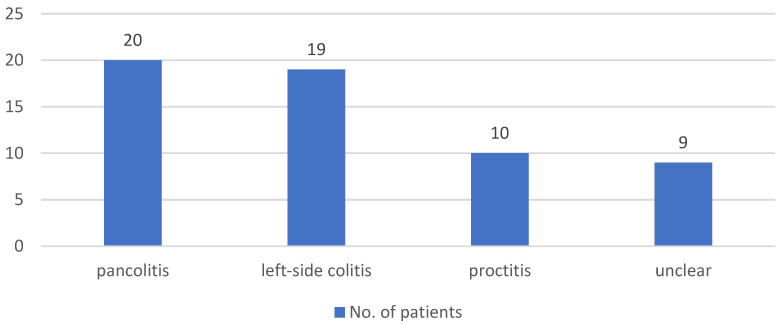
Distribution of disease patterns in UC patients in the study cohort.

A total of 7 out of 134 (5.2%) patients had a stoma at the time of the study. On average, patients had been hospitalized twice due to their chronic inflammatory bowel disease since the initial diagnosis. The pattern of disease involvement was also analyzed, with Figure 1 showing that CD patients were primarily affected in the small intestine (including the ileocecal valve), followed by involvement of both the small and large intestine. In patients with ulcerative colitis, the initial pattern of involvement was mostly pancolitis, followed by left-sided colitis and, finally, proctitis (see Figure 2).

**Figure 3:**
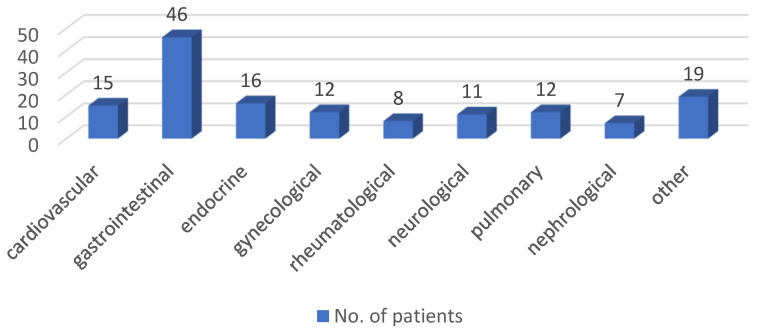
Presence of comorbidities in all IBD patients who had children, highlighting common gastrointestinal and cardiovascular conditions in the cohort.

Furthermore, we analyzed comorbidities in patients who had children, with gastrointestinal comorbidities being the most common, including conditions such as gastritis, reflux esophagitis, and liver or biliary tract diseases. Cardiovascular disease was the second most common comorbidity in our patient cohort. These and other comorbidities are detailed in Figure 3.

### 3.3. Medication

Within the study, we analyzed the IBD medications for all 134 patients. It is worth noting that patients with an uncomplicated disease course and no need for specific treatment were less likely to be seen at our university hospital outpatient clinic. The most frequently used medications were 5-Aminosalicylic Acid (5-ASA), followed by TNF-alpha blockers. Systemic glucocorticoids were mostly used for short-term flare-up therapy. Some patients had undergone long-term steroid treatment before being referred to our outpatient clinic, with the goal of establishing a steroid-free treatment plan.

Table 3 details the most commonly used medications by the IBD patients, along with treatment response and duration (in months), excluding medications used during pregnancy or breastfeeding.

### 3.4. Pregnancy

In this cohort analysis, patients were retrospectively screened and analyzed between January 2021 and December 2021, while the prospective questionnaire was administered over a six-month period beginning in May 2023.

At the time of conducting the patient survey, 3 out of 80 (3.8%) female patients were pregnant. Of the 80 included female patients who had children, data on pregnancy outcomes could be analyzed for 40 women; furthermore, 23 out of 40 (57.5%) surveyed patients consulted a doctor for pre-pregnancy advice.

As it is crucial to achieve disease remission prior to planning pregnancy, the focus was on maintaining remission for at least three months before conception.

Of the surveyed patients, 34 out of 40 (85.0%) were in remission for at least three months before conception. One of the surveyed patients had received fertility treatments.

**Figure 4:**
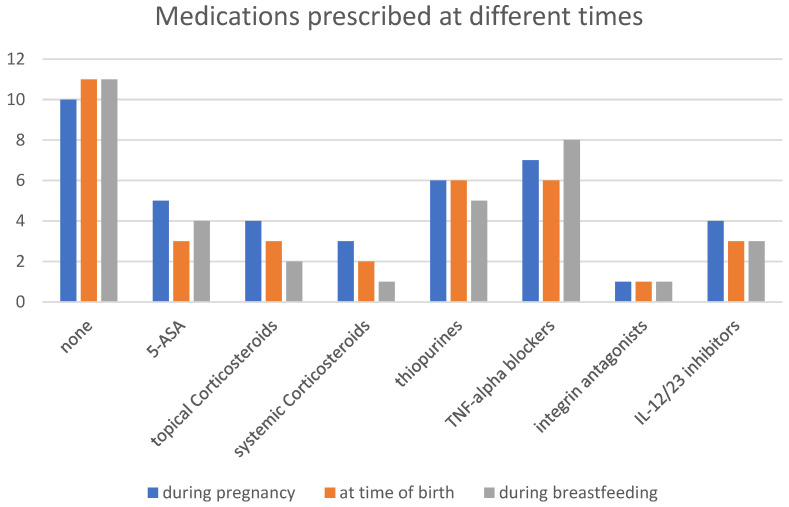
Overview of prescribed medications for surveyed IBD patients used during pregnancy, at the time of birth, and during breastfeeding.

An overview of the pharmaceutical treatments during pregnancy, at the time of birth, and during breastfeeding is detailed in Figure 4.

TNF-alpha blockers were most commonly used during pregnancy. Due to acute IBD flares, systemic glucocorticoids were temporarily prescribed for 3 out of 39 patients, with initial doses ranging between 20 and 30 mg. The dosages were subsequently tapered and discontinued over the course of treatment. 5-ASA was also used as maintenance therapy in part of the patient cohort. None of the surveyed patients discontinued their ongoing medication on their own.

**Figure 5:**
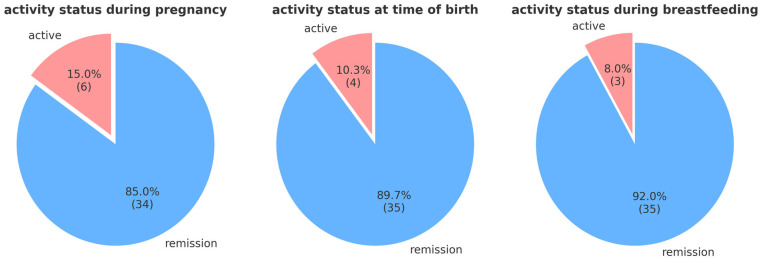
IBD activity at different timepoints (during pregnancy, birth, and breastfeeding) for surveyed female patients with IBD.

At the time of conception, 6 out of 40 patients (15.0%) experienced an active IBD flare. By the time of delivery, 4 out of 40 patients (10.0%) were not in remission. During breastfeeding, 3 out of 39 patients (7.7%) were not in remission (see Figure 5).

Regarding other parameters during pregnancy, 20% of patients exhibited anemia, which was managed with iron, vitamin B12, or folate supplementation as required. The median CRP value at the time of pregnancy was 0.78 mg/dL (range 0.08–5.54). The median fecal calprotectin value was 77.5 µg/g (range 33.6–228). Pregnancy showed no significant effect on the change in fecal calprotectin levels.

Only 12.5% of the patients experienced gestational diabetes. Due to side effects (pancreatitis), IBD treatment (azathioprine) was adjusted for one patient during pregnancy. Ultrasound examinations and amniotic fluid levels were unremarkable in 21 out of 23 (91.3%) surveyed patients during pregnancy. There were no cases of pre-eclampsia or premature placental detachment within the included patient cohort.

We analyzed the disease activity status during pregnancy, at the time of birth, and during breastfeeding. We found that 14.6% (*n* = 6) of the patients experienced at least one acute flare during pregnancy. At the time of birth, the proportion was 10.3% (*n* = 4) and, during breastfeeding, it was 7.2% (*n* = 3).

### 3.5. Pregnancy Outcomes

#### 3.5.1. Delivery Method

The proportion of women who underwent cesarean section was 30% (12/40), comparable to the cesarean rate in Germany among non-IBD patients (32.1%) [17]. Anesthesia administered included 60% general anesthesia and 40% epidural (see Table 4). Within the patient cohort, 92.5% (*n* = 37) of the surveyed women experienced no complications during birth. One patient (1/40, 2.5%) suffered stalled labor, and another experienced placental insufficiency. In both cases, the women were in remission. However, the woman who experienced stalled labor had a pouch. One patient had an abortion for personal reasons. The specific reasons for cesarean sections in the majority of cases were not sufficiently documented or explored in this study.

#### 3.5.2. Outcomes (Newborns)

The median gestation period was 38.9 weeks (range 37–43). The median birth weight was 3005 g (range 1830–4000). The median height of newborns was 50.1 cm (range 1830–4000). Two newborns suffered from pneumonia at birth. Otherwise, the remaining children (38/40, 95%) were born healthy. Routine 4-week pediatric examination (U3) showed no abnormalities in any of the children (see Table 5).

#### 3.5.3. Breastfeeding

Breastfeeding data were available for a total of 21 included female patients, with 81% (17/21) able to breastfeed within the first hour. None of the mothers were advised against breastfeeding. The median duration of breastfeeding was 5 months. The survey and retrospective data analysis revealed that 3 out of 56 children of IBD patients showed gastrointestinal symptoms, such as diarrhea (see Table 6).

### 3.6. Outcomes in Men

The evaluation of outcomes for the children was possible for 32 men. None of the surveyed male patients reported that the birth of their children was associated with complications or that the children exhibited gastrointestinal symptoms (see Table 7).

### 3.7. Experience About Pregnancy Counseling

The survey included an open question regarding how well women with IBD felt supported by their physicians in navigating pregnancy. Only nine patients provided responses to this question. Seven of them felt well advised and had no further concerns or worries about pregnancy complications related to their disease activity. In two cases, patients were erroneously advised against pregnancy by their former physicians.

### 3.8. Correlation Between Remission Before Pregnancy and Pregnancy Complications

After analyzing all retrospective and prospective data from women with IBD who had children during their disease, we investigated the correlation between remission prior to pregnancy (at least three months before conception) and pregnancy complications. None of the women in either group (in remission or not) experienced pre-eclampsia. The duration of pregnancy was also not significantly different between the groups.

It is important to note that the group of patients with active disease was small. In Table 8, we outline various parameters regarding pregnancy outcomes in the IBD patients.

The total number of respondents varies across different metrics in this table. This variation occurs because not all surveyed patients answered every question. Such differences in response rates are common in surveys and reflect the voluntary participation of respondents.

We analyzed the correlations between remission prior to pregnancy and pregnancy complications (see Table 9). Gestational diabetes occurred more frequently in patients with active disease than in those in remission (25.0% vs. 10%). Additionally, patients with active disease experienced fever during pregnancy more often than patients in remission (25% vs. 5.0%).

## 4. Discussion

In our study, we analyzed pregnancies and pregnancy-related complications in patients with IBD in relation to disease activity, within a cohort attending a high-level care facility. Our cohort analysis showed that effective therapy before and during pregnancy is essential for a complication-free pregnancy and healthy child outcomes. Additionally, our analysis revealed consistent pregnancy outcomes within our cohort, supported by recent studies indicating that effective management is essential for positive pregnancy outcomes [19].

### 4.1. Medical Safety During Pregnancy

Furthermore, proper medical consultation before conception is crucial to alleviate patient concerns about complications and the exacerbation of inflammatory bowel disease (IBD) symptoms. Not only is adequate IBD medication important, but lifestyle changes also play a significant role. Several studies have demonstrated that smoking is a significant risk factor for pregnancy complications in patients with IBD [21,22].

It is also important that IBD therapy continues during pregnancy; furthermore, breastfeeding should not be discontinued without consulting the treating physician. During routine consultations in our clinic, we advised against discontinuing medications during pregnancy without prior consultation [23,24].

Several studies have shown that administering mesalamine during pregnancy does not increase the risk of pregnancy complications, when compared to untreated IBD patients or non-IBD patients [24,25].

Clinical data on TNF-alpha inhibitors, such as infliximab or adalimumab, suggest their safety during pregnancy, with no associated adverse pregnancy outcomes having been observed [11]. These findings offer reassurance to both patients and healthcare providers concerning the safety profile of TNF-alpha inhibitors when used during pregnancy in women with IBD [26].

Several studies have argued that conventional steroid therapy does not adversely affect pregnancy. However, attention should be paid to the placental passage of specific steroids, such as prednisolone. On the other hand, budesonide, which is commonly used in treating IBD, is minimally absorbed from the intestine into the bloodstream, making it safer during pregnancy [27,28,29].

Vedolizumab and Ustekinumab are safe during pregnancy, with minimal adverse effects and lower drug levels in newborns. Final doses are recommended 8–12 weeks before delivery, and breastfeeding is likely safe due to low milk concentrations. For Ustekinumab, live vaccines should be delayed for a year post-birth, unless the drug is cleared from the infant [30]. In addition, Ustekinumab has shown high efficacy, particularly in patients with CD who have therapy-resistant disease activity [31]. Pregnancy outcomes with Ustekinumab are comparable to those without IBD [32].

The CESAME study indicated that IBD patients who received thiopurines during pregnancy did not have a significantly increased risk of preterm birth, low birth weight, or congenital abnormalities, compared to those receiving other therapies or no therapy at all [33].

JAK inhibitors, including tofacitinib and filgotinib, are contraindicated during pregnancy according to the 2022 ECCO guidelines, and should be discontinued before conception [34].

Pregnancy should be avoided in patients receiving S1P modulators, such as ozanimod, and for at least 3 months after discontinuation. Effective contraception is strongly recommended during ozanimod treatment [35].

In general, except for methotrexate, JAK inhibitors, sphingosine 1-phosphate (S1P) modulators, and thalidomide, most IBD medications are regarded as safe and well tolerated during pregnancy, without an increased risk of complications [36,37]. Notably, our study provides evidence supporting this overall safety profile with concrete data on the favorable tolerability of the previously mentioned medications. Within our study group, medications including 5-ASA, topical corticosteroids, thiopurines, TNF-alpha blockers, integrin antagonists, and IL-12/23 inhibitors were primarily administered during pregnancy and breastfeeding. These treatments exhibited no adverse events or pregnancy-associated complications, emphasizing their safety for use in this specific patient population [38]. As noted, well-controlled disease activity (clinical remission) is the most critical prognostic factor for an uncomplicated pregnancy.

### 4.2. Pregnancy Complications

Our study revealed no significant correlations between pregnancy complications and inflammatory bowel disease when the disease activity is well managed (i.e., in remission). The outcomes for the children of mothers with IBD in remission before and during pregnancy were not significantly worse, when compared to data for mothers without IBD, although it was noted that these children had comparatively lower birth weights. Breastfeeding was also uncomplicated, and only 5.3% of the respondents (2 out of 38) reported IBD-typical symptoms in children.

### 4.3. Surgical History

IBD patients with a history of surgical interventions might experience a more complicated course regarding family planning. For example, several studies have shown that IBD patients with an ileoanal pouch have an increased risk of infertility—necessitating fertility treatments for those desiring children—and may also be more prone to pregnancy complications [39]. In general, the literature shows that cesarean sections are more common in IBD patients, when compared to the general population [40]. Additionally, a cesarean section is strongly recommended for cases of active perianal Crohn’s disease, while ileal pouch surgery may be a potential consideration for a cesarean section [36,37]. In our study, one woman who had an ileoanal pouch experienced stalled labor, illustrating the potential complications that can arise during pregnancy in patients with a surgical history.

Miscarriage was reported in only one case, which was due to personal reasons. Several studies have been unable to determine whether chronic inflammatory bowel diseases pose a risk factor for miscarriage. According to Mahadevan et al., there were no significant differences in the rates of therapeutic abortions and congenital anomalies between groups, nor in the frequency of congenital anomalies among children whose mothers suffered from UC or CD [41].

In our study, we observed a decrease in the number of pregnant IBD patients with active disease as pregnancy progressed (six patients during pregnancy and three during lactation). A similar finding has been reported by van der Giessen et al., who found a significant decrease in proinflammatory cytokines with advancing pregnancy. This supports the hypothesis that pregnancy is safe and potentially beneficial for IBD patients, in terms of disease activity [42].

### 4.4. Patient Education and Support

The ECCO guidelines also confirm our hypothesis that uncomplicated pregnancy is possible for IBD patients. The diagnosis of IBD during pregnancy introduces additional concerns and anxiety, which are addressed through collaborative efforts in monitoring and treating the disease during this life stage. Achieving and maintaining disease remission is crucial for a successful and uneventful pregnancy [43].

Furthermore, the data concerning male participants should not be overlooked. Men with IBD also experience concerns about the potential impact of their disease on their partner’s pregnancy and the health of their offspring. This underscores the importance of involving both partners in counseling sessions to ensure that men receive adequate information and support regarding family planning and, thus, can confidently pursue parenthood despite their condition. Given that family planning choices are often regarded as private, they are not always openly discussed in physician–patient interactions. It is essential to actively address this topic in physician–patient interactions to improve patient decision making and experiences regarding pregnancy. It is critical that IBD patients be informed of the potential risks associated with discontinuing their medications without medical advice in order to ensure that remission is maintained [44].

With regard to post-onset IBD, the literature indicates that pregnancy outcomes in patients with post-onset IBD are comparable to those of patients with a pre-pregnancy diagnosis of IBD [45].

The survey results highlight that, despite the risk of disease exacerbation during pregnancy, informed counseling and support from healthcare professionals can significantly alleviate patient concerns and support their decision to pursue pregnancy.

### 4.5. Limitations and Future Research

It should be noted that not all study participants fully completed the questionnaire during the collection of prospective data, and some patients did not provide any information regarding the course of their pregnancy or the condition of their children. As a result, it can be noted that the total numbers for some variables in the included tables vary from other variables.

Additionally, retrospective data could not be completely collected due to patients discontinuing treatment at our center (e.g., for reasons such as relocation). Nevertheless, the available data were carefully analyzed to conduct as meaningful an analysis as possible.

Further prospective studies in larger cohorts are needed to analyze and define the correlations between pregnancy and chronic inflammatory bowel diseases in order to identify which patients should be considered at higher risk regarding pregnancy more precisely.

## 5. Conclusions

Patients with chronic inflammatory bowel diseases can safely become pregnant, provided that they are in remission before and during pregnancy, as seen in our cohort. The survey results highlight the importance of achieving remission prior to conception, as patients in this state tended to experience fewer complications. In contrast, those with active disease faced a higher likelihood of issues such as gestational diabetes and fever during pregnancy. A professional doctor–patient relationship and consultation are essential to alleviate patient concerns. If disease activity persists during pregnancy planning, it is advisable to postpone pregnancy until stable remission is achieved.

## Figures and Tables

**Table 1:** Study population and clinical presentation of all the study participants who had children (prospective and retrospective) including age, diagnosis, remission status, and family history.

	Females	Males
number of patients	80 (59.7%)	54 (40.3%)
age (median)	65.5 (range 46–85)	54.5 (range 27–67)
diagnosis (CD:UC:IBD-U)	42 (52.5%):33 (41.2%):5 (6.3%)	27 (50.0%):26 (48.1%):1 (1.9%)
age at initial diagnosis, median	33.5 (range 17–77)	33 (range 9–75)
illness duration until pregnancy	7 (range 3–21) years	4 (range 2–10) years
surgical intervention	25/80 (31.3%)	20/54 (37.0%)
clinical remission *	73/80 (91.25%)	36/54 (66.7%)
family history of IBD	9/80 (11.25%)	5/54 (9.3%)
allergies	33/80 (41.3%)	3/54 (5.6%)
weight (median), in kg	68 (range 43–92)	81 (range 67–111)
smoking	4/80 (5%)	1/54 (1.9%)

Abbreviations: CD, Crohn’s disease; UC, ulcerative colitis; IBD-U, inflammatory bowel disease unclassified; IBD, inflammatory bowel disease. * Under therapy at the time of data collection.

**Table 2:** Surgical interventions of all patients who had children, detailing types of procedures performed and their respective frequencies within the study cohort.

Surgery Type	Number of Patients with Surgery	%
surgical abscess drainage	13/134	9.7%
ileocecal resection	24/134	17.9%
small bowel resection	6/134	4.5%
hemicolectomy	8/134	6.0%
total colectomy	4/134	3.0%
revision or adhesiolysis	3/134	2.2%
sigmoid resection	1/134	0.7%
stoma	7/134	5.2%
ileoanal pouch	16/134	12.0%

**Table 3:** IBD medication statistics at the time of data collection for all IBD patients who had children, including response rates and average duration of therapy.

Medication	No. of Patients	Treatment Response	Duration of Therapy (in Months) in Months (Average)
5-ASA (oral and rectal)	82	53 (64.6%)	33.5 (range 2–288)
topical glucocorticoids	63	35 (55.5%)	22.7 (2–288)
systemic steroids	70	49 (70.0%)	19.9 (2–96)
thiopurine	58	34 (58.6%)	38.1 (2–120)
calcineurin antagonist	2	2 (100%)	22.0 (2–42)
methotrexate	8	2 (25.0%)	31.0 (3–180)
TNF-alpha blockers	75	60 (80%)	34.5 (2–120)
integrin antagonists	41	33 (80.4%)	20.2 (3–60)
interleukin-12/23 antibodies	37	28 (75.6%)	20.7 (3–68)
janus kinase inhibitors	5	3 (60.0%)	10.0 (3–18)

**Table 4:** Delivery methods and anesthesia types in female IBD patients, along with complication rates during childbirth.

delivery method	spontaneous: 20/40 (62.5%) C-section: 12/40 (37.5%)
anesthesia	general: 9/15 (60.0%), epidural 6/15 (40.0%)
complications	none: 37/40 (93.8%), stalled labor: 1/40 (2.5%) placental insufficiency: 1/40 (2.5%)

**Table 5:** Outcomes for newborns of IBD patients, including birth weight, gestational age, and any complications.

gestational age at birth (median)	39 weeks (range 37–43)
birth weight (median)	3005 g (range 1830–4000)
birth height (median)	50.1 cm (range 41–55)
child abnormalities	none: 38/40 (95.0%), pneumonia: 2/40 (5.0%)
pediatric check-up	normal: 40/40 (100.0%)

**Table 6:** Details on breastfeeding and IBD symptoms in children of patients with IBD, including counseling provided and breastfeeding duration.

breastfeeding within the first hour possible	17/21 (81.0%)
breastfeeding counseling (1: Yes, 2: No)	17/21 (81.0%)
breastfeeding duration (median)	5 months (range 1–13)
discouraged from breastfeeding	0/21 (0.0%)
gastrointestinal symptoms in children	2/38 (5.3%)

**Table 7:** Pregnancy outcomes for partners of male IBD patients, including preterm birth and miscarriage rates.

Outcome	Preterm Frequency
preterm birth	0/32 (0.0%)
miscarriage	0/32 (0.0%)

**Table 8:** Pregnancy outcomes in IBD patients: detailed examination of clinical and laboratory parameters including gestational diabetes, CRP levels, and ultrasound findings.

Characteristic	Frequency
current pregnancy	3/80 (3.8%)
fertility treatment	1/80 (1.3%)
consulted a doctor for advice	23/40 (57.5%)
in remission at least 3 months before pregnancy	34/40 (85.0%)
fever during pregnancy	2/24 (8.3%)
complaints during pregnancy (diarrhea, abdominal pain)	8/40 (20.0%)
flare during pregnancy	6/40 (15.0%)
self-discontinued therapy during pregnancy	0/40 (0.0%)
therapy discontinued due to side effects	1/40 (2.5%)
anemia	3/15 (20.0%)
CRP (median), mg/dL	0.78 mg/dL (range 0.08–5.54)
fecal calprotectin (median), µg/g	80 µg/g (range 33.6–600)
gestational diabetes	3/24 (12.5%)
duration of pregnancy (median), in weeks	39 (37–40)
sonography during pregnancy	normal in 21/23 (91.3%)
amniotic fluid volume	normal in 21/23 (91.3%)
pre-eclampsia	0/23 (0.0%)
premature detachment of placenta	0/23 (0.0%)
previous pregnancies	15/22 (68.2%)
steroid therapy during pregnancy	3/37 (8.1%)
vaccinations (COVID-19, influenza, whooping cough)	6/26 (23.1%)
changed lifestyle during pregnancy	34/40 (85.0%)
abortion (for personal reasons)	1/40 (2.5%)

**Table 9:** Analysis of correlation of pre-conception remission status with pregnancy complications in IBD patients, detailing the incidence of gestational diabetes, fever, and other pregnancy outcomes.

	In Remission Before Pregnancy				
	Yes (*n* = 34)		No (*n* = 6)		*p*-Value
pre-eclampsia	0/20	0.0%	0/4	0.0%	1.000
duration of pregnancy (median), in weeks	38.90 (SD = 1.51)		37.75 (SD = 0.83)		0.172
gestational diabetes	2/20	10.0%	1/4	25.0%	0.437
fever during pregnancy	1/20	5.0%	1/4	25.0%	0.437
ablatio placentae	0/20	0.0%	0	0.0%	1.000
birth weight	2983.57 (SD = 729.422)		3155.00 (572.756)		1.000
spontaneous delivery	16/30	53.3%	3/5	60.0%	1.000
cesarean section	11/30	36.7	1/5	20%	1.000

## Data Availability

All data generated or analyzed during this study are included in this article and its Supplementary Materials. Further inquiries can be directed to the corresponding author.

## Supplementary Materials

The following supporting information can be downloaded at: https://www.mdpi.com/article/10.3390/jcm13247710/s1. Supplementary material: English-translated prospective questionnaire; Table S1: parameters and scoring criteria of the Crohn’s Disease Activity Index (CDAI) for surveyed female patients with Crohn’s disease during pregnancy; Table S2: parameters and scoring criteria of the partial Mayo score for surveyed female patients with ulcerative colitis during pregnancy.

## Abbreviations

IBD	inflammatory bowel disease
CD	Crohn’s disease
UC	ulcerative colitis
IBD-U	inflammatory bowel disease unclassified
GI	gastrointestinal
TNF	tumor necrosis factor
JAK	janus kinase
5-ASA	5-aminosalicylic acid
CRP	C-reactive protein
