# Supplementary material for: Pregnancy in Inflammatory Bowel Disease: Data from a Real-World Cohort in Germany
Source: J Clin Med. 2024 Dec 17;13(24):7710. doi: 10.3390/jcm13247710 (PMC11678727; doi:10.3390/jcm13247710)
Supplement: Supplementary file 1 [file jcm-13-07710-s001.zip › jcm-3292342-supplementary.pdf]

UNIVERSITÄTSKLINIKUM AUGSBURG

Questionnaire number: ....

Table S1: Parameters and scoring criteria of the Crohn's Disease Activity Index (CDAI) for surveyed female patients with Crohn's disease during pregnancy.

**Parameter***	**Description**	**Results (median)**
weight	measured body weight, in kg	75.0 (range 60-92)
ideal body weight	using Devine formula based on sex and height (kg)	63.0 (51-70)
stool Frequency (x2)	number of soft/liquid stools in 7 days	10.0 (3-18)
abdominal pain (x5)	pain severity score in 7 days (rating, 0-3)	0.0 (0-2)
general well-being (x7)	self-reported well-being (rating, 1-4)	0.0 (0-1)
anti-diarrhea drug use (x30)	using anti-diarrheal medications	0.0 (0-1)
abdominal mass (x10)	no = 0, equivocal = 2, yes = 5	0.0 (0-2)
hematocrit (x6)	blood hematocrit level (%)	36.0 (28-45)
extraintestinal findings (x20)	arthritis/arthralgias, iritis/uveitis, erythema nodosum**, Anal fissure***, other fistula, fever > 37.8°C (1 point each × 20)	1 (0-2)
**CDAI**	**C**rohn's **D**isease **A**ctivity **I**ndex	**89.0 (54-228)**

*including the weighting factors, daily sum per week, **erythema nodosum, pyoderma gangrenosum, or apthous stomatitis, ***anal fissure, fistula, or abscess

Table S2: Parameters and scoring criteria of the Mayo score for surveyed female patients with ulcerative colitis during pregnancy.

**Parameter (rating 0-3)**	**Description**	**Results (median)**
stool frequency	number of bowel movements/day more than normal<br>**0**: normal, **+1**: 1-2 stools, **+2**: 3-4 stools, **+3**: >4 stools	1.0 (range 0-3)
rectal bleeding	**0**: none, **+1**: visible blood <50% of time, **+2**: ≥50%, **3**: blood only	0.0 (0-2)
mucosal appearance at endoscopy	**0**: normal, **+1**: mild, **+2**: severe	0.0 (0-2)
physician rating of disease activity	**0**: normal, **+1**: mild, **+2**: moderate, **+3**: severe	0.0 (0-3)
**Mayo Score (0-12)**		**1 (range 1-9)**

Questionnaire number: ....

Questionnaire: Pregnancy in Inflammatory Bowel Disease: Data from a Real-World Cohort in Germany

Thank you for your participation and support!
Please answer each question. Estimated information is better than no information.
I ask you not to be bothered if some aspects are asked several times from different perspectives.
Your data will be treated confidentially and will not be passed on to third parties or made accessible to others.

1. Which chronic inflammatory bowel disease have you been diagnosed with by your doctor?

☐ Crohn's disease ☐ Ulcerative colitis ☐ Inflammatory bowel disease unclassified (IBD-U)

2. When did your doctor first diagnose you with inflammatory bowel disease?

____

3. Involvement pattern:

Crohn's disease:
☐ small intestine (including terminal ileum)
☐ Colon
☐ Sigmoid Colon
☐ Rectum
☐ Upper digestive tract

Ulcerative colitis
☐ Left-sided colitis
☐ Pancolitis
☐ Rectum
☐ Backwash ileitis

4. Do any of your family members suffer from inflammatory bowel disease?

☐ No
☐ Yes

If yes:
☐ First-degree relatives (parents, siblings)
☐ Second-degree relatives (grandparents, aunts, uncles)
Type of inflammatory bowel disease:
☐ Crohn's disease
☐ Ulcerative colitis
☐ Inflammatory bowel disease unclassified (IBD-U)

Questionnaire number: ....

5. Do you suffer from any of the following conditions?

☐ Allergies to medication
☐ Heart disease
☐ Lung diseases
☐ Kidney disease
☐ Rheumatism (inflammatory joint diseases)
☐ Diabetes mellitus
☐ Thyroid disease
☐ Hepatitis B or C, HIV
☐ Anemia or other blood diseases
☐ Other autoimmune diseases. If yes, please specify: ________
☐ Other diseases, if yes, please specify: ________

6. Please list the medications you are currently taking or have taken, including the dosage and duration of use (if known). If a medication was discontinued, please select the reason from the list below.

Reasons for discontinuation:

1. Intolerance
2. Primary non-response
3. Secondary loss of efficacy
4. Regularly discontinued
5. Discontinued by physician
6. Other (Please specify): ____________

Please complete the following for each medication used:

Drug	Duration (from...to...)	Dose	Discountinued (Reason)
5-ASA (oral and rectal)			
topical glucocorticoids			
systemic steroids			
thiopurine			
calcineurin antagonist			
methotrexate			
Infliximab / Adalimumab			
Vedolizumab			
Ustekinumab			
Tofacitinib			
Other: _______			

7. All medications can have effects—some good (like helping your condition) and some not so good (like side effects). Are you currently experiencing, or have you ever experienced, any side effects from your IBD medication?

____

____

8. What is your marital status?

____

9. In the course of your chronic inflammatory bowel disease (IBD):

Have you ever been hospitalized for a flare-up of your IBD?

☐ No
☐ Yes, if yes, please provide details: When? How many times? For how long?

____

Questionnaire number: ....

Have you ever had surgery related to your IBD?

☐ No

☐ Yes, if yes, please specify: When? What type of surgery? Where was it performed?

____

10. Are you a member of an IBD support group or organization?

☐ No
☐ Yes, with the German Crohn's Disease/Ulcerative Colitis Association
☐ Yes, with another organization ______________________________

11. Do you have a pouch (a reservoir made from loops of the small intestine) or a stoma (an artificial bowel outlet)?

☐ No
☐ Yes, a pouch
☐ Yes, a stoma

12. Do you smoke, or does anyone in your household smoke?

☐ No

☐ Yes, if yes, please specify: How much and for how long? _________________

13. Do you drink alcohol regularly?

☐ No
☐ Yes, If yes, please specify: How much and since when?

14. Are you symptom-free with your current IBD therapy?

☐ Yes
☐ No, if No, please specify the IBD-related symptoms you currently experience:

____

15. Chronic inflammatory bowel disease (IBD) can be associated with various symptoms and related conditions. These may include abdominal pain, joint pain, fistulas, abscesses (in the abdominal or anal area), skin changes, eye inflammation, or fever over 38°C.
Have you developed any related conditions? If so, please specify:

____

____

16. Do you have children?

☐ No
☐ Yes, If yes, how many? Have any of your children been diagnosed with inflammatory bowel disease?

____

	Complaints	Age of diagnosis
1. Child		
2. Child		
3. Child		

Questionnaire number: ....

<u>The following questions are for female patients only. There are no additional questions for male patients.</u>

17. Could you currently be pregnant?

☐ No
☐ Yes, if yes, please specify the week of pregnancy: ________________

18. Pregnancy Course: (Please provide information about your current or most recent pregnancy)

<u>- Did you undergo fertility treatment?</u>
☐ No
☐ Yes, If yes, please specify the type (e.g., clomiphene, egg donation, IVF, artificial insemination):

____

- Were you symptom-free or in remission for at least three months before your pregnancy?
☐ Yes
☐ No, if No, what symptoms or complications occurred at that time?

____

- Did you consult a doctor for advice before your pregnancy?
☐ No
☐ Yes

- What medication were you taking at the time of pregnancy? Please specify the type and dosage:

____
____

- Were you hospitalized for your IBD within the three months before or during your pregnancy?
☐ No
☐ Yes, if yes, please specify the reason and duration of hospitalization:

____

- Which IBD medications did you take regularly during your pregnancy or your last pregnancy?

____
____

- If you took steroids during your pregnancy, what dosage and for how long?

____

- Did your doctor pause or change the dosage of any IBD medications during your pregnancy?
☐ No
☐ Yes, if yes, please specify which medications, when, or in which week of pregnancy:

____

- Reason for Discontinuation or Change of Therapy:
Please select the reason for any therapy discontinuation or change:
☐ Discontinued by my doctor before the start of my pregnancy
☐ Occurrence of complications during my pregnancy. If yes, what kind of complications?

____

Questionnaire number: ....

☐ I discontinued the medication without consulting my doctor

Reason: ____

- Did you take any other medication (apart from IBD medication) regularly during your pregnancy?

☐ No

☐ Yes, if yes, please specify: _________________________________

- Were you symptom-free regarding your IBD during your pregnancy?

☐ Yes

☐ No, if No, please describe any complications, complaints, or medication side-effects you experienced:

____

- How often did you have flare-ups, and what symptoms did you experience?

____

- During your pregnancy, did you try any alternative, complementary, or naturopathic treatments for your IBD (e.g., acupuncture, Ayurvedic medicine, homeopathy, traditional Chinese medicine)?

☐ No

☐ Yes, if yes, please specify: ____________________________________

- Ultrasound examination:

☐ Normal

☐ Abnormal, If abnormal, please provide details: ___________________

- Amniotic fluid volume:

☐ Normal

☐ Decreased

☐ Increased

- Fever > 38.5°C during pregnancy:

☐ No

☐ Yes

- Gestational diabetes:

☐ No

☐ Yes

- Pre-eclampsia (pregnancy-related high blood pressure):

☐ No

☐ Yes

- Placental abruption:

☐ No

☐ Yes

- Vaccinations during pregnancy:

☐ No

☐ Yes, If yes: Vaccine name: ________________________________

Questionnaire number: ....

Date of vaccination (week of pregnancy): ___________

Side-effects after vaccination:_____________________

- Have you changed your lifestyle during pregnancy?

☐ More exercise before and during pregnancy

☐ Avoided alcohol, smoking, and caffeine

☐ Taking supplements (e.g., folic acid…)

- If you answered "yes" to any of the questions or experienced other illnesses or complications not covered above, please provide further details:

____

____

19. Pregnancy outcome:

Planned date of birth or current week of pregnancy: ____

- Outcome:

☐ Live birth

☐ Miscarriage / Stillbirth

☐ Termination of pregnancy.   Date: ___________ Gestational week: _____

-> Reason for termination:

☐ Maternal illness

☐ Fetal abnormalities

☐ Personal reasons

☐ IBD-related complications (please specify): ______________________________

20. Birth:

- Delivery method:

☐ Live birth

☐ Miscarriage / Stillbirth

- Type of anesthesia:

☐ General anesthesia

☐ Regional anesthesia (e.g., epidural)

21. Child:

- Gender:

☐ Male

☐ Female

☐ Indeterminate

- Date of birth and gestational week at delivery: ______________________________

- Birth weight (g): ________________

- Height (cm): ___________

- Any abnormalities in the child?

☐ No

☐ Yes, If yes, specify and indicate when detected: ____________________________

Questionnaire number: ....

- Date of hospital discharge of the child: ___________________________________
- Routine 4-week pediatric examination (U3):

☐ Unremarkable
☐ Abnormal findings, if abnormal, please describe: ________________________
☐ Still pending

---

22. Breastfeeding:

- Were you able to breastfeed your child within the first hour after birth?

☐ No
☐ Yes

- Did you receive breastfeeding consultation?

☐ Detailed
☐ Briefly
☐ No

- How long did you breastfeed your child? ___ Months ___ Weeks

- Were you advised against breastfeeding by a healthcare professional?

☐ No
☐ Yes

- If you did not breastfeed, what was the reason?

☐ Concern about IBD relapses
☐ Required IBD medication due to flare-ups
☐ Other: _____________________________

- Have you noticed any of the following in your child?

☐ Allergic skin reactions (e.g., rashes, swelling, itching)
☐ Diarrhea
☐ Gas and abdominal pain
☐ Redness or soreness (e.g., diaper area)

- Did you experience IBD flare-ups while breastfeeding?

☐ No
☐ Yes, if yes, please specify frequency and type of symptoms: ____________

- Which IBD medication did you take while breastfeeding?

Medication and dosage: ____

- Did you stop any IBD medications while breastfeeding?

☐ No
☐ Yes, If yes, specify which medication(s): ____________________________________

-> Reason for discontinuation:

☐ I stopped taking my IBD medication without consulting my doctor
☐ My doctor advised me to stop taking the medication

---

23. Previous Pregnancies:

- Have you had previous pregnancies?

☐ No
☐ Yes, If yes

-> If yes, please specify the number of each:

* Healthy children born alive: ______________
* Children born alive with congenital conditions: ______________
* Miscarriages: ______________
* Ectopic pregnancies: ______________

Questionnaire number: ....

* Stillbirths: ____________
* Abortions (reason): _____________

- Details of previous pregnancies (e.g., medications during pregnancy, treatment discontinuation, and reasons for discontinuation):

____

____

24. Did you feel that you received adequate advice on managing pregnancy with IBD? Were you able to ask all your questions on this topic? Have you ever been advised against pregnancy by a doctor or others due to your IBD, despite pregnancy being possible for IBD patients?

____

____

**Thank you once again for your participation
and support of medical research!**
